# A Highly Active Endo-Levanase BT1760 of a Dominant Mammalian Gut Commensal *Bacteroides thetaiotaomicron* Cleaves Not Only Various Bacterial Levans, but Also Levan of Timothy Grass

**DOI:** 10.1371/journal.pone.0169989

**Published:** 2017-01-19

**Authors:** Karin Mardo, Triinu Visnapuu, Heiki Vija, Anneli Aasamets, Katrin Viigand, Tiina Alamäe

**Affiliations:** 1 Department of Genetics, Institute of Molecular and Cell Biology, University of Tartu, Tartu, Estonia; 2 Laboratory of Environmental Toxicology, National Institute of Chemical Physics and Biophysics, Tallinn, Estonia; National Renewable Energy Laboratory, UNITED STATES

## Abstract

*Bacteroides thetaiotaomicron*, an abundant commensal of the human gut, degrades numerous complex carbohydrates. Recently, it was reported to grow on a β-2,6-linked polyfructan levan produced by *Zymomonas mobilis* degrading the polymer into fructooligosaccharides (FOS) with a cell surface bound endo-levanase BT1760. The FOS are consumed by *B*. *thetaiotaomicron*, but also by other gut bacteria, including health-promoting bifidobacteria and lactobacilli. Here we characterize biochemical properties of BT1760, including the activity of BT1760 on six bacterial levans synthesized by the levansucrase Lsc3 of *Pseudomonas syringae* pv. tomato, its mutant Asp300Asn, levansucrases of *Zymomonas mobilis*, *Erwinia herbicola*, *Halomonas smyrnensis* as well as on levan isolated from timothy grass. For the first time a plant levan is shown as a perfect substrate for an endo-fructanase of a human gut bacterium. BT1760 degraded levans to FOS with degree of polymerization from 2 to 13. At optimal reaction conditions up to 1 g of FOS were produced per 1 mg of BT1760 protein. Low molecular weight (<60 kDa) levans, including timothy grass levan and levan synthesized from sucrose by the Lsc3Asp300Asn, were degraded most rapidly whilst levan produced by Lsc3 from raffinose least rapidly. BT1760 catalyzed finely at human body temperature (37°C) and in moderately acidic environment (pH 5–6) that is typical for the gut lumen. According to differential scanning fluorimetry, the T_m_ of the endo-levanase was 51.5°C. All tested levans were sufficiently stable in acidic conditions (pH 2.0) simulating the gastric environment. Therefore, levans of both bacterial and plant origin may serve as a prebiotic fiber for *B*. *thetaiotaomicron* and contribute to short-chain fatty acids synthesis by gut microbiota. In the genome of *Bacteroides xylanisolvens* of human origin a putative levan degradation locus was disclosed.

## Introduction

We are not alone. Billions of microorganisms live in and on our bodies. Among these microbes, the gut community comprises a major part and largely affects our well-being and health [1–2]. As quoted by Vrieze *et al*. [3], intestinal microbiota can be viewed as an exteriorised organ that contributes to overall metabolism and plays a role in converting food into nutrients and energy. The gut bacteria feed mostly on complex polysaccharides (food fiber, microbiota-accessible carbohydrates) that are resistant to degradation by gastric acid and host digestive enzymes. Resulting fermentation products, for example short-chain fatty acids (SCFA) and some other metabolites of microbial origin, have numerous beneficial functions for the host [1,4]. Certain poly- and oligosaccharides such as galactooligosaccharides, inulin (a β-2,1-linked fructan) and β-2,1-linked (inulin-type) fructooligosaccharides (FOS) are well-known and widely used prebiotics which specifically promote growth of the approved probiotic bacteria–lactobacilli and bifidobacteria [5–7]. However, rapid accumulation of new data on gut microbiota and its multiple functions has fueled search of prebiotics for other potentially beneficial gut bacteria such as *Faecalibacterium* and *Bacteroides* which are abundant commensals in healthy adults [8–11].

The human gut bacteroidetes bacteria belong to order *Bacteroidales* having three dominant genera: *Bacteroides*, *Parabacteroides* and *Prevotella*. Individual strains and species of *Bacteroidales* are highly abundant in the gut reaching densities of 10^9^–10^10^ colony-forming units per g of feces [12]. The *Bacteroides* genus stands out due to possession of impressive repertoire of polysaccharide-degrading enzymes [13–15] being thereby perfectly equipped for the consumption of nutrients available in the colon. For example, members of this genus degrade resistant starch, pectin, galactomannan, glucomannan, arabinogalactan, alginate, laminarin, xylan, β-glucan, rhamnogalactan and cellulose [14,16–17]. *Bacteroides thetaiotaomicron* which is numerous in the gut and is considered either a commensal or a symbiont has gained specific attention from scientific community [18–21]. Sonnenburg *et al*. [7] reported on the ability of *B*. *thetaiotaomicron* to grow on levan, a β-2,6-linked fructose polymer, and proved that the endo-levanase (BT1760; BT_1760) was indispensable for it. From other tested *Bacteroides* species (*B*. *caccae*, *B*. *ovatus*, *B*. *fragilis*, *B*. *vulgatus* and *B*. *uniformis*), only *B*. *thetaiotaomicron* grew on levan, the other tested species did not have a BT1760 homologue in their fructan utilization loci [7].

Levan-type fructans are mostly synthesized by bacterial enzymes [22], but are present also in some plants. For example, timothy grass (*Phleum pratense*) and orchard grass (*Dactylis glomerata*) have linear β-2,6-linked fructans which are referred to as phleins or plant levans [23–25]. Mixed levans (graminans) which contain both, β-2,1 and β-2,6 linkages, are present in many *Poales* species (e.g. ryegrass), but also in agave and in essential food cereals such as wheat, rye and barley [26–28]. Bacterial levan is currently produced by only a few companies such as Montana Polysaccharides Corp. (USA) and it is used in foods, beverages, medicine and nanotechnology [29–32]. Levan is permitted as functional food additive in Japan and South Korea [29] whereas it is currently not commercially produced and applied in Europe. According to our knowledge, levan-type FOS are not commercially manufactured. They have been produced at small scale for research using either acid-aided [33] or enzymatic [34] hydrolysis of bacterial levan.

We have enzymatically produced the FOS with degree of polymerization (DP) of 3–8 by reacting a highly active levansucrase Lsc3 of *Pseudomonas syringae* pv. tomato with sucrose in conditions that favour the FOS production [10,35]. After precipitation of polymeric levan from the reaction mixture, FOS, some fructose, a high amount of glucose (an inevitable by-product) and residual sucrose remain in the supernatant. In our approach, glucose and fructose were removed from the FOS solution using treatment with an invertase-negative *Saccharomyces cerevisiae* strain [10,35]. This additional step prolongs and complicates the FOS production procedure. Moreover, the FOS mixture synthesized by the levansucrase Lsc3, contained not only β-2,6-linked FOS, but also β-2,1-linked oligosaccharides, e.g. 1-kestose [10].

The aim of the current study was to conduct in-depth characterization of heterologously expressed endo-levanase BT1760 of *B*. *thetaiotaomicron* by addressing biochemical properties of the enzyme that were not studied in [7]. A variety of β-2,6-linked fructans–different reference levans of bacterial origin synthesized from sucrose, a levan synthesized from raffinose (Raf) by levansucrase Lsc3 of *P*. *syringae*, and a plant levan (from timothy grass)–were assayed as substrates of the endo-levanase. We show that the BT1760 can be applied for efficient production of FOS from levans. Prebiotic potency of these FOS can then be studied using bacterial pure cultures as well as gut consortia. Levan-derived FOS contain practically only β-2,6 linkages and can be used without further purification if minor amounts of fructose do not interfere with the assay. Stability of the levans and FOS at acidic conditions simulating the gastric environment and resistance to heat sterilization by autoclaving were also tested. Additionally, we compared the sequences of various fructan-degrading enzymes and screened the databanks for BT1760 homologues.

## Materials and Methods

**Fig 1:**
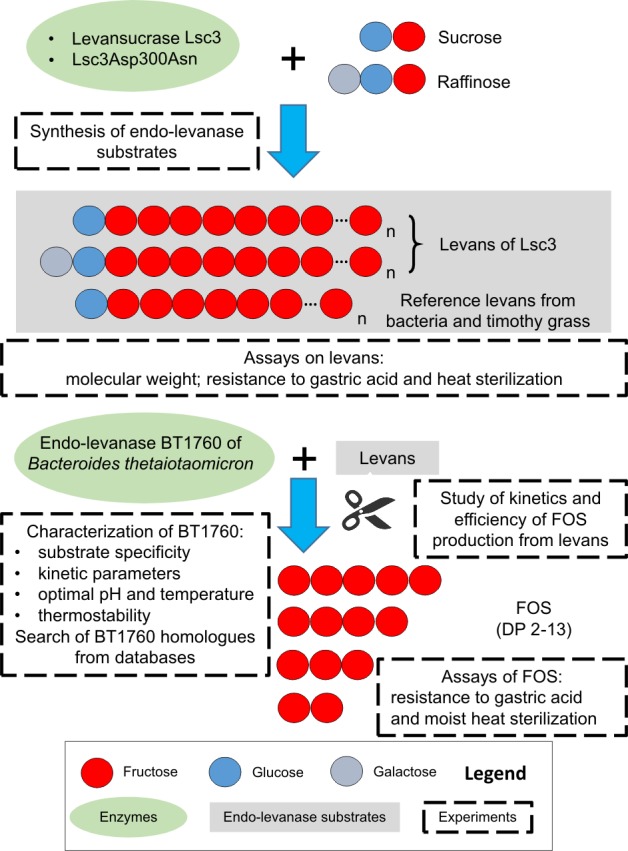
Scheme of the experiments. Degree of polymerization (DP) of levans designated as ‘n’ can be very high–over 10 000 [31]. For simplicity, levans are shown unbranched and fructooligosaccharides (FOS) derived from endo-levanase reaction are shown to contain only fructose.

Overwiew of experimental setup is presented in Fig 1.

### Levans and other sugars used in the assays

Seven different levans were used in this study: 1) levan synthesized from 1.2 M (410.8 g/L) sucrose by levansucrase Lsc3 of *Pseudomonas syringae* pv. tomato; 2) levan synthesized from 0.3 M (151.3 g/L) raffinose by Lsc3 of *P*. *syringae*; 3) levan synthesized from 1.2 M (410.8 g/L) sucrose by the Lsc3Asp300Asn (D300N) mutant of *P*. *syringae*; 4) levan of *Zymomonas mobilis* 113S (kindly provided by Dr. A. Vigants, University of Latvia, Latvia); 5) levan (L8647) *of Erwinia herbicola* (*Pantoea agglomerans*) from Sigma-Aldrich (Germany); 6) levan from *Halomonas smyrnensis* (a gift from Prof. E.T. Öner, Marmara University, Turkey; isolated as in [36]); 7) levan from timothy grass (a gift from Dr. A. Kasperowicz, Polish Academy of Sciences, Poland; isolated as in [37]). Levans of *Z*. *mobilis*, *E*. *herbicola* and *H*. *smyrnensis* are all produced from sucrose. 1-kestose, nystose, stachyose and inulin from dahlia were from Sigma-Aldrich (Germany), FOS-preparations P95 and Synergy1 were from Beneo (Belgium), xylooligosaccharide mixture was from Sweet Town Biotech (Taiwan) and raffinose was from Naxo (Estonia).

### Synthesis and purification of levans. Enzymatic production of fructooligosaccharides (FOS) from levan

Levan synthesis using Lsc3 and Lsc3Asp300Asn from 1.2 M sucrose was carried out as described in [10]. For purification of levans from reducing/low molecular weight sugars, they were dissolved at 50–100 g/L in sterile mQ water and dialysed using Servapor dialysis membrane (MWCO 12–14 kDa; Serva, Germany) in the case of Lsc3-produced levan or Spectra/Por membrane (MWCO 3.5 kDa; Spectrum Laboratories, USA) in the case of Lsc3Asp300Asn-produced levan. Levans were dialysed against sterile mQ water containing 0.02% of sodium azide at 4°C and vacuum-dried thereafter [10]. Levan from 0.3 M raffinose was produced as in [10] using the Lsc3 protein, precipitated by alkaline ethanol in the presence of 0.4% NaCl and dialysed as described above. Raffinose was used at lower concentration compared to sucrose due to its reduced solubility. Purity of levan preparations was confirmed by measuring reducing sugar content in a dinitrosalicylic acid (DNSA) assay [38] and absence of FOS in the levan preparations was verified using thin layer chromatography (TLC) (described below).

FOS were produced from Lsc3-synthesized high molecular weight (HMW) levan as follows. 50 g/L of levan in 25 mM Na-phosphate buffer (pH 6.0) was reacted at 37°C during 6 h in sterile conditions with 16 mg/L (3 U/mL) of heterologously produced endo-levanase BT1760 (see below). Reaction was stopped by heating (96°C; 5 min) and fructose was removed from the preparation by a 48 h-treatment with yeast *Ogataea* (*Hansenula*) *polymorpha* HP201 [39] similarly as in the case of treatment with baker’s yeast [10,35]. Yeast-treated FOS preparation was filter-sterilized and vacuum-dried [10].

### Characterization of levans: assay of molecular weight, resistance to heat sterilization and hydrolysis by 0.01 M HCl

Levans synthesized by Lsc3 and Lsc3Asp300Asn from 1.2 M sucrose were analysed for average molecular weight in the laboratory of Prof. A. López-Munguía (UNAM, Cuernavaca, Mexico) by high-performance size-exclusion chromatography (HPSEC) on UltiMate 3000 Rapid Separation eqipment (Dionex, Thermo Scientific, USA) coupled with refractive index detector (Shodex, Germany). Two Ultrahydrogel SEC columns were set up in series (Ultrahydrogel Linear, 7.8 × 300 mm, and Ultrahydrogel 500, 7.8 × 300 mm, Waters, USA) using 0.1 M NaNO_3_ as eluent at flow rate of 0.8 mL/min at 30°C. Commercial dextrans of 5.2–5000 kDa (Waters, USA) were used as calibration standards. Molecular weight data of levans of *Zymomonas mobilis*, *Erwinia herbicola*, *Halomonas smyrnensis* and of timothy grass phlein were received from from the literature and the providers.

To assess acid-tolerance of the fructans, levans, dahlia inulin (all used at 10 g/L) and FOS mixture (13 g/L) derived from levan by endo-levanase treatment (see above) were incubated in 0.01 M hydrochloric acid (pH 2.0) at 37°C for 24 hours. At certain time points samples were withdrawn and combined with two volumes of 0.01 M NaOH to stop the hydrolysis reaction. To achieve full hydrolysis of the fructans to monosaccharides, the samples were incubated at 37°C for 24 h in 0.2 M HCl, and the reaction was stopped by adding two volumes of 0.2 M NaOH. Reducing sugar content of latter samples was used as a reference (100%) to evaluate proportion of fructan hydrolysis at a certain time point.

Stability of the preparations to heat sterilization in the autoclave was studied using 0.5 mL samples of above-mentioned fructans in mQ water. The samples were sterilized at 112°C or 121°C for 15 min. Before and after the treatment, the samples were analysed for the hydrolysis products using TLC (see below) and for the reducing sugar content in a DNSA assay [38].

### Assay of long-term stability of the levansucrase Lsc3

Pure preparation of the levansucrase Lsc3 (Lsc-3; UniProtKB Q88BN6; GenBank AAO59056.1) of *Pseudomonas syringae* pv. tomato, produced as in [40], was assayed for long-term stability (S1 Fig). The enzyme was incubated at 37°C for over a year. At regular intervals, enzymatic activity of the preparation was measured at 37°C as in [40]. Protein content was determined according to optical density at 280 nm.

#### Bacterial strains and cultivation

Cloning of the BT1760 gene, heterologous expression and purification of the His-tagged endo-levanase BT1760. Assay of levan degradation by recombinant *Escherichia coli*

*E*. *coli* DH5α was used in cloning procedures and *E*. *coli* BL21(DE3) [41] as a host for endo-levanase expression. The transformants were grown on LB medium with ampicillin (0.15 g/L). Heterologous expression of endo-levanase was conducted as in [40]. Liquid cultures were aerated on a shaker. Growth temperature of bacteria was 37°C if not stated otherwise.

The endo-levanase BT1760 (UniProtKB Q8A6W6; GenBank AAO76867.1) was expressed without the predicted N-terminal signal peptide. First, the BT1760 sequence was amplified from genomic DNA of *Bacteroides thetaiotaomicron* VPI-5482 (DSM 2079; the DNA kindly provided by Prof. Reet Mändar, University of Tartu, Estonia) using Pfu polymerase (Thermo Scientific, USA) and primers BT1760_Fw_EcoRI (5´TAAAGAATTCAGTGACGAGACTGACCCCATCTTG3´) and BT1760_Rev_HindIII (5´TTTAAGCTTGCCGGTGTAGTTTTC 3´), the respective restriction sites are underlined. The resulting 1547 bp fragment was cloned into the pJET vector using CloneJET PCR Cloning Kit from Thermo Scientific (USA) yielding pJET-BT1760 (4521 bp). To produce the BT1760 with a C-terminal His_6_-tag, pURI3-BT1760Cter (3965 bp) was constructed as follows: BT1760 sequence was amplified from pJET-BT1760 with primers BT1760_PURI3Cter_Fw (5´**TAACTTTAAGAAGGAGATATACAT*****ATG****GACGAGACTGACCCCATCTTG*3´) and BT1760_PURI3Cter_Rev (5´**GCTA****TTA****ATGATGATGATGATGATG***ATAAGTGCTTACCTGAACGTCTG*3´). In the primers, the nucleotides annealing with the pURI3Cter vector [42] are shown in bold and those annealing with BT1760 sequence are shown in italics. The ATG start codon and the stop codon are underlined in the primers. The PCR product was cloned to pURI3Cter expression vector in a PCR-based/ligase-free procedure as in [40].

The pURI3-BT1760Cter was electroporated into *E*. *coli* BL21(DE3) and the expression, purification, dialysis and concentration of the His_6_-tagged endo-levanase was performed as in [40]. Expression level of the protein and purity of the preparation was assayed using SDS-PAGE (S2 Fig). Protein concentration in *E*. *coli* lysates was determined as in [43], in case of purified protein, the absorbance at 280 nm was measured and protein concentration in the preparation was calculated according to the extinction coefficient (see below).

To verify levan degradation by endo-levanase-expressing *E*. *coli*, 5 μL of overnight liquid culture harbouring pURI3-BT1760Cter was spotted onto LB + Amp plates containing 0.5 mM IPTG and 5 g/L levan and incubated overnight at room temperature. To study the state of levan degradation around and under the bacterial growth zone, small agar plugs were excised from the agar plate, melted at 100°C and assayed for levan degradation products using TLC.

### Assay of enzymatic activity and substrate specificity of the endo-levanase BT1760. Study of the effects of pH and temperature

Endo-levanase activity on above-mentioned fructans was routinely measured at 37°C in McIllvaine’s buffer (pH 6.0) containing 0.02% Na-azide [40]. Different fructans and other potential endo-levanase substrates were used at 5 g/L if not stated otherwise. Initial rate of fructan degradation was recorded by measuring the reducing sugar release in a DNSA assay [38] using fructose as a standard. One unit of endo-levanase activity was defined as the amount of enzyme that released 1 μmol of reducing sugar equivalents per minute. Specific endo-levanase activity was expressed in U per mg of protein. Activity of LevB of *Bacillus licheniformis* [34] on Lsc3-produced HMW levan (10 g/L) was assayed in 50 mM phosphate buffer (pH 6.0) as in Porras-Domínguez *et al*. [34] recording initial velocity of the reducing sugar release in a DNSA assay.

Kinetic parameters (K_m_, V_max_, *k*_cat_ and *k*_cat_/K_m_) of levan degradation by BT1760 were calculated on six levans (levan of *Erwinia herbicola* was excluded) by recording initial velocity of reducing sugar release at varied substrate concentrations (1–15 g/L). The data were analyzed using Enzyme Kinetics Module 1.1 of the Sigma Plot 2001 (Systat Software Inc., USA). Catalytic constants (*k*_cat_; 1/s) were calculated using V_max_ and theoretical molecular weight of the purified protein.

To monitor the kinetics and product spectrum of endo-levanase reaction, six different levans (5 g/L) were incubated at 37°C in McIllvaine’s buffer (pH 6.0) containing 0.02% Na-azide during 72 h. Samples withdrawn at desired time points were heated 5 min at 96°C to stop the reaction and analysed using high-performance liquid chromatography (HPLC) and TLC (see below). To eliminate subtle differences between the levan concentrations due to initial water content variations of the preparations, the HPLC results were normalized according to total saccharide concentration of the samples determined after complete levan degradation at 72 h. Levan conversion rate to FOS was calculated as percentage of FOS produced from 5 g/L of levan (taken as 100%) under the applied conditions. FOS yield was expressed as mg of FOS produced per mg of protein and it was calculated from FOS content (g/L) determined in the samples withdrawn at respective time points.

Temperature and pH dependence of endo-levanase activity was studied in a DNSA assay by measuring initial velocity of the reducing sugar release from 5 g/L of HMW levan produced by Lsc3 from sucrose. Protein content in the reaction mixtures was 4.6–11 μg/mL. The effect of pH was evaluated by conducting the reaction in McIllvaine’s buffer of varied pH (pH 3.5–8.0) at 37°C. The effect of temperature was evaluated at 30–60°C at pH 6.0. The protein was equilibrated for 5 min at respective conditions prior to the measurement.

For the assay of thermal stability of the enzyme, the endo-levanase was incubated in McIllvaine’s buffer (pH 6.0) at varied temperatures (30–60°C) for 30 minutes and thereafter cooled on ice. Residual endo-levanase activity of the samples was then measured at 37°C in a DNSA assay. Alternatively, thermal stability of the endo-levanase was evaluated using differential scanning fluorimetry as in Mardo *et al*. [44].

### Chromatography of levan degradation products

TLC of levan degradation products was performed on plates with concentrating zone (ALUGRAM® Xtra SILUGR UV_254_, Macherey-Nagel, Germany). The mixtures were spotted onto a TLC plate alongside with reference sugars. Chromatograms were developed twice with a solvent system of chloroform: acetic acid: water (60:70:10; v/v/v) or chloroform: methanol: water (90:65:15, v/v/v) [45–47]. The fructose-containing sugars were visualized by dipping the plate into a solution of 3% (w/v) urea and 1 M phosphoric acid in water-saturated butanol, and subsequent heating at 120°C for 10 min [48].

Mono- and oligosaccharides were quantified from the reaction mixtures essentially as in Mardo *et al*. [44] and Adamberg *et al*. [10] using HPLC by Acquity UPLC system (Waters, USA) and Alltech Prevail Carbohydrate ES column (Grace, USA) coupled with evaporative light scattering detector.

### *In silico* methods

SignalP program [49] at http://www.cbs.dtu.dk/services/SignalP/ was used to predict presence and cleavage site of the N-terminal signal sequence. ExPASy Proteomics Server (http://expasy.org) was used to calculate theoretical Mw and extinction coefficient at 280 nm of C-terminally His-tagged BT1760 for determination of protein concentration. Protein and genomic DNA sequences were searched and withdrawn using web-based programs and databases (NCBI BLAST, NCBI GenBank, EMBL-EBI, UniProtKB). Clustal Omega program [50] was used for the alignment and identity level analysis of protein sequences. Database accession numbers of protein and DNA sequences used in this work are presented in Supporting Information (S1 Table). BioEdit [51] and pDRAW32 software (http://www.acaclone.com/) were used to visualize the alignments and structure of the genomic loci, respectively.

## Results and Discussion

### Endo-levanase BT1760 among glycoside hydrolases, analysis of levanase protein sequences and genomic loci

Here, we analyse and compare the amino acid sequence of BT1760 with sequences of experimentally studied and putative endo- and exo-acting fructanases. Database accession numbers of the sequences addressed by us are presented in S1 Table of Supporting Information.

**Fig 2:**
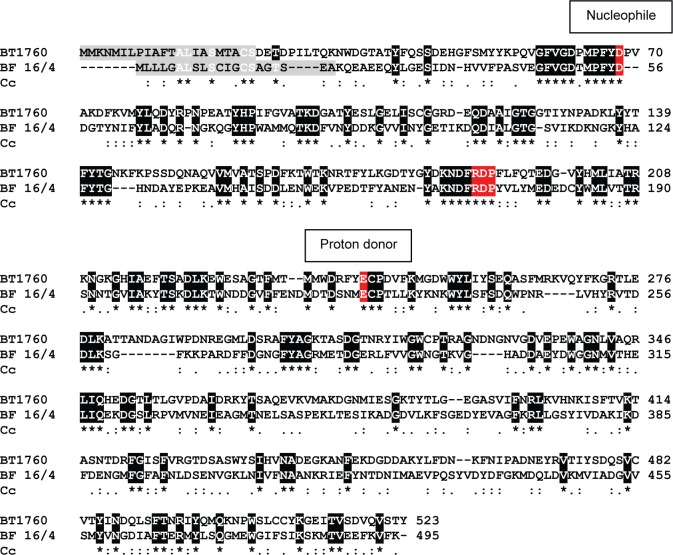
Clustal Omega alignment of the *Bacteroides thetaiotaomicron* endo-levanase BT1760 with putative levanase (D4IW69) of *Butyrivibrio fibrisolvens* 16/4 (BF 16/4). Identical residues between the sequences are shown on black background and marked with an asterisk. Similar residues are designated below the alignment with dots. Predicted N-terminal signal peptides are shown on grey background. Predicted nucleophiles, proton donors and the ‘RDP’ motifs are shown on red background. Cc–Clustal consensus.

CAZy database [52] defines endo-levanases (EC 3.2.1.65) as enzymes that perform random hydrolysis of β-2,6 fructofuranosyl linkages in β-2,6 fructans (levans) containing more than 3 fructose units [53]. Endo-levanases are grouped into Glycoside Hydrolase (GH) family 32 alongside with invertases (EC 3.2.1.26), endo-inulinases (EC 3.2.1.7), exo-inulinases (EC 3.2.1.80) and few other enzymes of somewhat similar sequence motifs and topology. These enzymes have a 5-bladed β-propeller fold and they hydrolyze glycosidic substrates by using a proton donor (a glutamate) and a nucleophile (aspartate in the most cases) in the catalysis [52]. No crystal structure for an endo-levanase is currently available in the PDB database. However the structure of the endo-inulinase INU2 of a filamentous fungus *Aspergillus ficuum* (PDB: 3RWK) has been resolved [54]. We aligned the sequences of INU2 and endo-levanase BT1760 of *Bacteroides thetaiotaomicron* and disclosed only a low (20.7%) overall identity between these proteins. Yet, the ‘RDP’ motif conserved in GH32 enzymes participating in substrate binding was detected in both, BT1760 (Fig 2) and INU2 [54]. The region next to the nucleophile (Glu43 in INU2 and Asp62 in BT1760) was poorly conserved between the enzymes: YWMN**E**PNG in INU2 and GFVG**D**PMP in BT1760, the nucleophiles are designated in bold. The sequence regions around the proton donor Glu233 in INU2 (Glu242 in BT1760) had higher identity: **E**CPDV in BT1760 and **E**VPDM in INU2, the proton donor is shown in bold. For the location of these predicted catalytic residues of BT1760, see Fig 2.

Multiple sequence alignment of experimentally studied endo-levanases revealed only modest identity with BT1760. For example the identity of the endo-levanase LevB1 of *Bacillus licheniformis* [34] with BT1760 was 21.3% and that of the endo-levanase of *Bacillus subtilis* 168 [55] even lower–only 18.3%. An endo-levanase of a rumen bacterium *Butyrivibrio fibrisolvens* 3071 has also been biochemically characterized [25], but respective gene and protein sequences are not available. We then used the BLASTP program at NCBI (https://www.ncbi.nlm.nih.gov/) to search potential endo-levanase proteins in a human fecal isolate *Butyrivibrio fibrisolvens* 16/4 [56]. With BT1760 sequence as a query, we retrieved a protein (D4IW69; annotated as levanase/invertase) with 31.6% identity to BT1760, whilst only 19.3% identity to LevB1 of *Bacillus licheniformis*. Identity values of the putative levanase of *B*. *fibrisolvens* 16/4 with exo-acting fructanases of *B*. *thetaiotaomicron* were between 19.6% (BT3082) and 21.6% (BT1765). Fig 2 shows alignment of the BT1760 and the putative levanase of *B*. *fibrisolvens* 16/4. Though these two proteins align well over the entire sequence length and share conserved regions, it does not fully confirm that D4IW69 of *B*. *fibrisolvens* 16/4 is an endo-levanase. As discussed in [34], sequence identity of a characterized endo-levanase (from *Bacillus subtilis*) with characterized exo-acting levanases (from *Streptomyces exfoliatus* and *Microbacterium laevaniformans*) can be high–up to 60%. Thus, high overall sequence identity is not sufficient to discriminate between the endo- and exo-acting levanases, thereby biochemical studies of respective enzymes are required.

**Fig 3:**
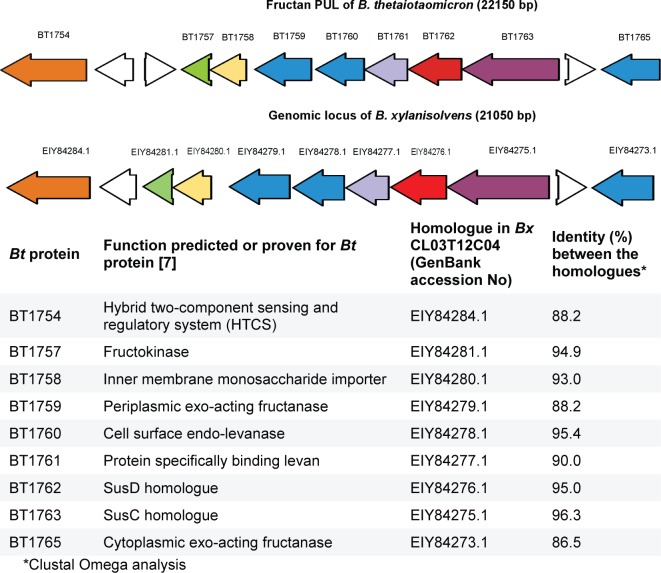
Comparison of fructan PUL of *Bacteroides thetaiotaomicron* (*Bt*) VPI-5482 [7] with genomic locus of *Bacteroides xylanisolvens* (*Bx*) CL03T12C04 harboring a close homologue of the *B*. *thetaiotaomicron* endo-levanase BT1760. Colour code is used to designate homologous proteins of the two loci. ORFs of levan-hydrolyzing proteins of *B*. *thetaiotaomicron* and their homologues in *B*. *xylanisolvens* are in blue. Intervening unrelated genes [7] are in white.

According to [7], the fructan PUL (polysaccharide utilization locus) of *B*. *thetaiotaomicron* encodes not only the endo-levanase BT1760, but also several exo-acting fructanases. So, BT1759, BT1765 and BT3082 are exo-fuctanases producing fructose from both, levan and inulin, whereas BT1760 is a levan-specific endo-fructanase (endo-levanase) [7]. Fig 3 illustrates organisation of respective genes in the fructan PUL of *B*. *thetaiotaomicron*. We compared the PUL-encoded proteins (database accession numbers are given in S1 Table) by sequence alignment and detected a quite high (53.8%) identity between BT1765 and BT1759. These two proteins had lower, approximately 35% sequence identity with BT3082. Notably, BT3082 is encoded outside the fructan PUL [7]. Identity of the BT1760 (endo-levanase) with three exo-acting fructanases of *B*. *thetaiotaomicron* (BT1759, BT1765 and BT3082) was from 18.7% (BT3082) to 20.3% (BT1759).

We then searched homologues of BT1760 from sequence databases using the BLASTP program. Proteins with high (over 90%) sequence identity to BT1760 were detected among translated genomic sequences of many *Bacteroides* species. For example, a hypothetical glycosyl hydrolase EIY84278.1 of *Bacteroides xylanisolvens* CL03T12C04, a strain of human origin, had 95.4% identity with BT1760. We retrieved and analysed the genomic region of *B*. *xylanisolvens* CL03T12C04 harbouring this putative endo-levanase gene. Data in Fig 3 suggest that the genomic region of *B*. *xylanisolvens* CL03T12C04 containing the putative endo-levanase gene is a fructan PUL that is similar to that of *B*. *thetaiotaomicron* [7]. Aside of endo-levanase proteins, the SusD and SusC homologues of these two PULs have very high sequence identities. Importantly, the SusD-like protein BT1762 is also levan-specific–it binds levan at the cell surface and has no affinity for inulin. When respective gene was deleted from *B*. *thetaiotaomicron*, growth of the bacterium on levan was strongly retarded [7]. BT1761 is a putative outer membrane lipoprotein that also binds levan, but not inulin [7]. BT1761 had 90% sequence identity with its predicted homologue in *Bacteroides xylanisolvens* (Fig 3). We hypothesize that *Bacteroides xylanisolvens* CL03T12C04 may also catabolize levan and produce FOS.

### Synthesis of levan from sucrose and raffinose using levansucrase Lsc3 and characterization of levans used in the current study

Levansucrase Lsc3 (Lsc-3) of *Pseudomonas syringae* pv. tomato and its mutant Asp300Asn [40] were used in this work to produce levans as substrates for the endo-levanase BT1760 (see Fig 1). The Lsc3 has a very high catalytic activity–the *k*_cat_ for sucrose-splitting is over 500 1/s and the yield of levan in a 20-h reaction with 1.2 M sucrose is up to 13 g per 1 mg of Lsc3 protein [22]. Therefore, it is certainly feasible for biotechnological applications. Lsc3 is also intriguingly stable at storage. Long-term stability monitoring of Lsc3 revealed that for over 200 days of storage at 37°C, the residual activity of the enzyme did not decrease (S1 Fig).

Though mostly a disaccharide sucrose (α-Glc-1,2-β-Fru) is used as a substrate for levan and FOS synthesis, bacterial levansucrases, including those of *P*. *syringae* can also use a trisaccharide raffinose (α-Gal-1,6-α-Glc-1,2-β-Fru) [47,57–60]. As levans produced from sucrose and raffinose may have different properties [57], we synthesized levans from both of these sugars (see Fig 1). Gel permeation chromatography (HPSEC) of levan synthesized by Lsc3 from sucrose revealed a bi-modal size-distribution of polymer chains: it contained a HMW fraction of approximately 4700 kDa and a LMW fraction of approximately 11 kDa (Table 1). The HMW fraction (see Materials and Methods and Table 1) was used as endo-levanase substrate in the current work if not stated otherwise. A HMW (3500 kDa) and a LMW (8.3 kDa) fractions have also been described in levan produced by *Bacillus subtilis* 168 [61]. A LMW levan (Table 1) used in this work was prepared from sucrose using the Asp300Asn (D300N) mutant of Lsc3 [40,44] which was uncapable of HMW levan synthesis. The data including sources and molecular weights of timothy grass levan and bacterial levans produced from sucrose are presented in Table 1.

**Table 1:** Size-distribution of levans used in the study.

**Levan**	**Average molecular weight (kDa)**	**Origin/Reference/Remarks**
Lsc3* **levan from 1.2 M sucrose (before the dialysis)**	4733 ± 125 (HMW); 10.7 ± 1.0 (LMW)	Current work
Lsc3* **levan from 1.2 M sucrose (after the dialysis)**	4760 ± 158 (HMW)	Current work. This HMW levan was used as a substrate in characterization of BT1760 properties, if not stated otherwise.
Lsc3D300N* **levan from 1.2 M sucrose**	16.6 ± 0.5; 7.4 ± 0.1	Current work
***Erwinia herbicola* levan**	1100–1600	[62]
***Zymomonas mobilis* levan**	~2000	A. Vigants, personal communication
***Halomonas smyrnensis* levan**	1483	[63]
**Timothy grass levan**	~60	[25,64–65]

Average molecular weight and standard deviation values of three independent measurements are shown.

* Levan synthesized by *Pseudomonas syringae* pv. tomato levansucrase Lsc3 or its mutant D300N. Levans of *E*. *herbicola*, *Z*. *mobilis* and *H*. *smyrnensis* are synthesized from sucrose (see Materials and Methods).

### Heterologous expression of endo-levanase BT1760 in *Escherichia coli* and initial assessment of its activity

Similarly to endo-inulinase INU2 of *Aspergillus ficuum* and other GH32 enzymes [54], endo-levanase BT1760 of *Bacteroides thetaiotaomicron* is predicted to have a bi-modular arrangement: in addition to N-terminal catalytic β-propeller domain, a C-terminal β-sandwich domain is found which may function in either substrate binding or protein stability. We cloned and produced the BT1760 with a C-terminal His_6_-tag as a two-domain full-length protein excluding the leader peptide (see Materials and Methods). SignalP program predicted cleavage of the leader peptide from the BT1760 between the Ser22 and Asp23 residues. Calculated molecular weight of the expressed protein was 57716 Da.

**Fig 4:**
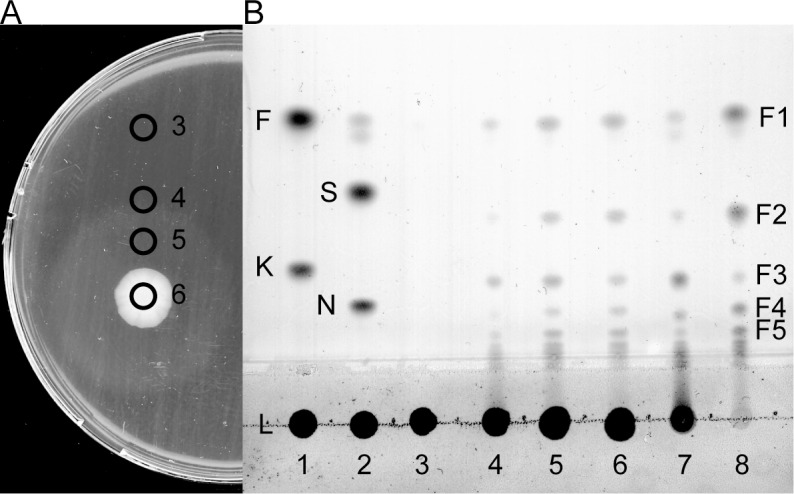
Levan degradation by *Escherichia coli* expressing endo-levanase BT1760 of *Bacteroides thetaiotaomicron* from the pURI3-BT1760Cter plasmid. (A) 5 μL of the liquid culture of recombinant *E*. *coli* was spotted onto a LB plate containing Amp, IPTG and 5 g/L of Lsc3-produced HMW levan and grown overnight at room temperature. Agar discs were cut off from regions marked with black rings, melted by heating and 0.5 μL of the sample was applied to a thin layer chromatography (TLC) plate. (B) TLC analysis of levan degradation from regions 3–6 of the agar plate in panel A and on lanes 7 and 8 products from 5 g/L levan by 1 h and 4 days of reaction with the lysate from recombinant *E*. *coli*. The chromatogram was developed in chloroform: methanol: water (90:65:15, v/v/v). Reference sugars on the TLC plate: fructose (F) and 1-kestose (K) on lane 1; sucrose (S) and nystose (N) on lane 2. F1-F5; levan degradation products with respective degree of polymerization.

First we assayed the endo-levanase activity in lysates of *Escherichia coli* overexpressing the endo-levanase BT1760. When the lysate was incubated with 5 g/L HMW levan synthesized by Lsc3, reducing sugars were produced at a rate of 90.6 ± 10.1 μmol/mg x min (U/mg) indicating perfect expression level of the enzyme which was confirmed by SDS-PAGE (S2 Fig). Notably, FOS and fructose are both reducing sugars, whereas levan is not. Degradation of levan to FOS by *E*. *coli* lysate can be seen on lanes 7 and 8 of Fig 4B. If a small portion (5 μL) of the liquid culture of a BT1760-expressing transformant was spotted onto LB plates containing Amp, IPTG and 5 g/L HMW levan and incubated overnight at room temperature, a wide turbid zone emerged around the growth area of the bacteria (Fig 4A). When agar discs were cut off from different regions of a plate (designated on Fig 4A by black circles), melted and analysed for levan degradation products, FOS were revealed in samples taken from the turbid zone or at its border (discs 6, 5 and 4 in Fig 4A and lanes 6, 5 and 4 in Fig 4B, respectively). At a distant zone of the plate, levan stayed undegraded (disc 3 in Fig 4A and lane 3 in Fig 4B). Turbidity zone seen on the agar plate is most probably caused by degradation of polymeric levan to FOS. This experiment highlights the possibility of high-throughput screening of bacterial strains or libraries of mutated enzymes for endo-levanase activity. Simultaneously to levan degradation, the DP range of produced FOS could be followed if the samples are analysed on TLC. We later repeated the experiment by using the purified endo-levanase instead of levanase-expressing *E*. *coli* culture. When 5 μL (approximately 20 μg) of purified endo-levanase protein were spotted on an agar plate containing 5 g/L of Lsc3-produced levan, a clear zone of levan hydrolysis became visible after overnight incubation of the plate at room temperature indicating complete hydrolysis of levan and longer FOS species. Again, a turbid zone was visible in the vicinity of a clear central zone.

### Effects of pH and temperature on catalytic activity of the endo-levanase BT1760

In these studies, HMW levan synthesized by Lsc3 from sucrose was used as a substrate for BT1760. First, we measured release of reducing sugars by BT1760 from 5 g/L of levan in McIllvaine’s buffer of varied pH. According to our results, the enzyme had a moderately wide pH optimum: at pH 4.5–6.5 the enzyme exhibited over 80% of its maximum activity. However, the activity was highest at pH 5.5–6.0 which was also confirmed in a levan degradation assay (S3 Fig). According to the literature, the endo-levanases from *Bacillus licheniformis* [34] and *Butyrivibrio fibrisolvens* [25] were assayed at pH 6.0 and the *Bacillus* sp. endo-levanase at pH 5.5 [66]. The endo-levanase LevB from *Bacillus subtilis* 168 had an activity optimum in the pH range of 6.0–6.5 [55]. However, the endo-levanase BT1760 has been assayed [7] in a moderately alkaline buffer (20 mM Tris-HCl, pH 8.0). We consider that as the BT1760 locates on the cell surface of *Bacteroides thetaiotaomicron* [7], it should tolerate the acidic pH in the gut lumen resulting from fermentation of carbohydrates to short-chain fatty acids. So, Bondarenko *et al*. [67] showed that when levan-coated Co-nanoparticles were incubated with *B*. *thetaiotaomicron*, the metal was solubilized as the medium was acidified by levan fermentation products. At levan fermentation by *B*. *thetaiotaomicron* in an isothermal calorimeter, the pH decreased by about 1.5 units, dropping to pH 5.4–5.5 [10] which corresponds to active working pH of the enzyme.

**Fig 5:**
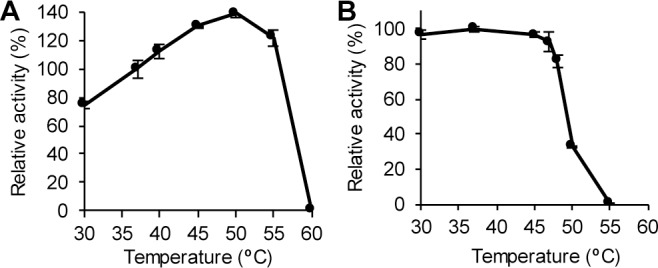
The effect of temperature on catalytic activity of the endo-levanase BT1760. (A) Temperature optimum of BT1760. Release of reducing sugars from levan by BT1760 was recorded at varied temperatures and relative activities were calculated. 100% of activity corresponds to 123.6 ± 6.4 U/mg, measured at 37°C. (B) Thermostability of BT1760. The endo-levanase BT1760 was incubated at indicated temperatures for 30 min, and residual endo-levanase activity was then measured at 37°C by recording reducing sugar release from levan. 100% of activity corresponds to 124.0 ± 1.8 U/mg. The average and standard deviation values are calculated form at least two independent measurements with two parallel samples analysed. Detailed description of the methods is presented in Materials and Methods.

The temperature optimum of the endo-levanase was 50°C. At higher temperatures the activity decreased sharply dropping to zero at 60°C (Fig 5A). Thermal inactivation assay showed that incubation of BT1760 at 50°C for 30 min strongly decreased its activity (Fig 5B). Thus, the enzyme can effectively catalyse at 50°C for a limited time (up to 5 min), whereas at longer incubation it will be inactivated. According to differential scanning fluorimetry, the T_m_ of the endo-levanase was 51.5°C. When compared with T_m_ (65.4°C) of the levansucrase Lsc3 of *Pseudomonas syringae* pv. tomato [44] that was applied in this study for levan synthesis, the respective T_m_ of BT1760 was considerably lower being comparable with respective value of the maltase protein of a yeast *Ogataea polymorpha* [68]. Notably, Lsc3 of *P*. *syringae* and BT1760 of *B*. *thetaiotaomicron* are both extracellular enzymes whereas the maltase is intracellular. *P*. *syringae* colonizes plant surfaces and its secreted enzymes should thereby resist various environmental stresses including a high temperature [22]. As *B*. *thetaiotaomicron* is a colon inhabitant, its extracellular enzymes must not tolerate high temperature. Indeed, at internal temperature of the human body (37°C), the endo-levanase BT1760 has perfect stability and activity (Fig 5). In our further experiments, the endo-levanase reaction was conducted in McIllvaine’s buffer of pH 6.0 at 37°C.

### Bacterial levans and a plant levan (phlein) are substrates for the endo-levanase BT1760, inulin and inulin-type fructooligosaccharides are not degraded

**Fig 6:**
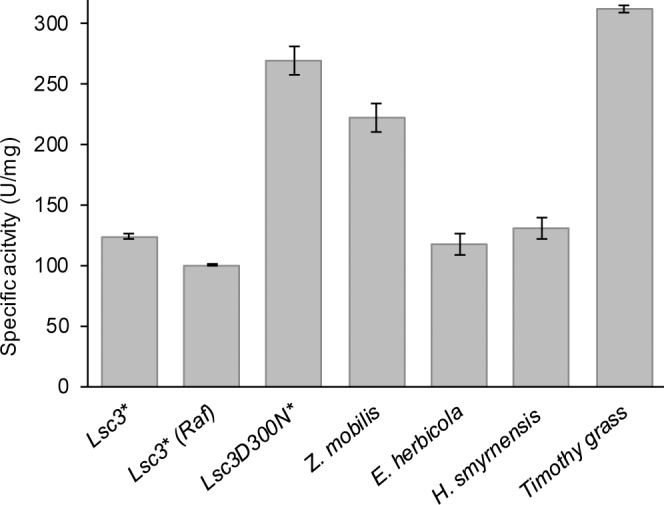
Initial velocities of reducing sugar release by the endo-levanase BT1760 from various levans added at 5 g/L. * Synthesized by *Pseudomonas syringae* pv. tomato levansucrase Lsc3 or its mutant Asp300Asn (D300N) from sucrose or raffinose (Raf). Mean values and standard deviation were calculated from at least three independent experiments. For additional information on levans, see Table 1.

Seven different levans were used for preliminary evaluation of substrate preference of BT1760 by measuring initial velocity of reducing sugar release from levans. Fig 6 shows that the timothy grass levan was degraded with the highest velocity. From bacterial levans, the Lsc3Asp300Asn-produced levan served as the best substrate, the *Z*. *mobilis* levan scoring as the second best (Fig 6). Levan synthesized from raffinose occurred as the least preferred substrate.

To obtain more detailed information on substrate preference of BT1760, kinetic characteristics–K_m_ and *k*cat−were determined for BT1760 catalysis on six levans, omitting the *Erwinia herbicola* levan (Table 2).

**Table 2:** Kinetic constants of endo-levanase BT1760 at degradation of various levans calculated from initial velocities of reducing sugar release.

Levan	*k*_cat_ (1/s)	K_m_ (g/L)	*k*_cat_/K_m_ (1/s x g/L)
Lsc3*	466.8 ± 27.7	13.6 ± 1.5	34.3
Lsc3* **(Raf)**	366.7 ± 23.3	12.2 ± 1.5	30.1
Lsc3D300N*	472.7 ± 16.8	4.1 ± 0.4	115.3
***Zymomonas mobilis***	442.9 ± 20.2	5.4 ± 0.5	82.0
***Halomonas smyrnensis***	333.0 ± 26.4	7.8 ± 1.3	42.7
**Timothy grass**	901.1 ± 57.3	7.7 ± 1.4	79.7

* Synthesized by *Pseudomonas syringae* pv. tomato levansucrase Lsc3 or its mutant Asp300Asn (D300N) from sucrose or raffinose (Raf). Mean values and standard deviations of at least three independent determinations are presented. For additional information on levans, see Table 1.

According to the K_m_, the most preferred levans for BT1760 were those produced by Lsc3Asp300Asn and *Zymomonas mobilis*, followed by levans of timothy grass and *Halomonas smyrnensis* (Table 2). All these levans are of low or moderarate molecular weight (Table 1). Affinity of BT1760 was the lowest for levan synthesized by the wild-type Lsc3 from sucrose. This levan has extremely high molecular weight–around 4.7 MDa (Table 1). Levan synthesized by Lsc3Asp300Asn was the best substrate for BT1760 also considering catalytic efficiency, followed by levans of *Z*. *mobilis* and timothy grass. Catalytic efficiency was the lowest for levans synthesized by wild-type Lsc3 from sucrose and raffinose. Sonnenburg *et al*. [7] studied catalytic activity of BT1760 using levan of *Z*. *mobilis* (Sigma; 66674, currently L8647). Kinetic constants for BT1760 obtained in [7] were close to those recorded by us in the case of *Z*. *mobilis* 113S levan. The difference between the K_m_ values of BT1760 measured on *Z*. *mobilis* levans (5.4 g/L by us versus 10.6 g/L by Sonnenburg *et al*. [7]) is probably caused by slightly dissimilar properties of these two levan preparations.

LevB1 protein of *Bacillus licheniformis* [34] is one of most thoroughly characterized endo-levanases. We had possibility to compare activities of LevB1 and BT1760 on HMW levan produced from sucrose by Lsc3. Endo-levanase reaction was conducted as in [34]: 10 g/L levan was used as a substrate and initial velocity of the reducing sugar release was measured. Surprisingly, BT1760 degraded the Lsc3-produced HMW levan about 300 times more rapidly than LevB1. The LevB was reported to hydrolyse a HMW levan, e.g. that of *Leuconostoc mesenteroides* NRRL-B512, rather poorly–with tenfold less activity than a LMW (8.3 kDa) levan of *Bacillus subtilis* [34]. Our data (Fig 6, Table 2) show that BT1760 certainly prefers LMW levans (of timothy grass and Lsc3Asp300Asn mutant) to those of moderate or high molecular weight, but the difference is not that large. We assume that compared to endo-levanase (LevB) of *Bacillus licheniformis*, the endo-levanase BT1760 of *Bacteroides thetaiotaomicron* is better suited for the hydrolysis of HMW levans.

BT1760 did not degrade dahlia inulin, xylooligosaccharides, raffinose and stachyose, and had only negligible activity on commercial inulin-type FOS preparations P95 and Synergy1. Earlier it has been shown that the BT1760 did not degrade chicory inulin, sucrose and inulin-type FOS, i.e. 1-kestose, nystose and fructosylnystose [7]. Inability to cleave sucrose, 1-kestose, 6-kestose and inulin (HP Orafti DP60) was also shown for endo-levanase LevB1 of *Bacillus licheniformis* [34].

We conclude that the BT1760 endo-levanase uses bacterial levans and levan of timothy grass as substrates, whereas it does not cleave inulin-type fructans. Though LMW levans are hydrolysed more rapidly than HMW ones, the HMW levans are still palatable substrates for BT1760. Also, considering endo-levanases studied so far, the BT1760 has the highest activity and catalytic efficiency on levans.

### Degradation of bacterial and plant levans to fructooligosaccharides (FOS) using endo-levanase BT1760: evaluation of the process

Endo-levanase can be used to efficiently produce β-2,6-linked FOS from levan as was earlier shown for the *Bacillus licheniformis* endo-levanase LevB1. When the latter enzyme was applied to a LMW levan (Prof. A. López-Munguía, personal communication) the reaction resulted in FOS of DP 2–8 with yield as high as 97%. At longer reaction, levanbiose was detected as the main hydrolysis product. The authors used the obtained FOS to evaluate the growth of probiotic bacteria [34].

**Fig 7:**
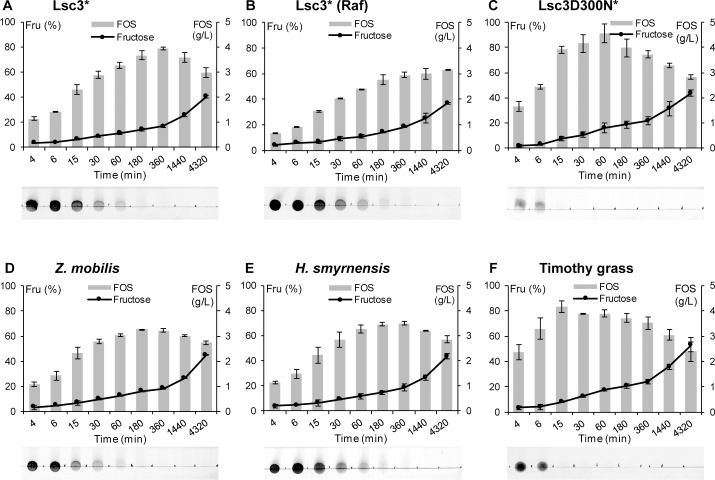
**Time course of degradation of six different levans by BT1760 (A-F).** Levans synthesized by Lsc3 or its mutant Asp300Asn (D300N) are designated by an asterisk. Degradation of the substrate (levan) is illustrated by the start region of developed thin layer chromatography plates inserted below each graph. Release of fructose presented as a line diagramme shows the percentage of fructose from total sugars detected in the sample during 72 h (4320 min) of the reaction. Total amount of FOS (g/L; DP from 2 to 13) produced by each time point is presented as grey bars. Up to four parallel samples were analysed to calculate the average values and standard deviations. Details of the reaction conditions and used methods are given in Materials and Methods section.

**Fig 8:**
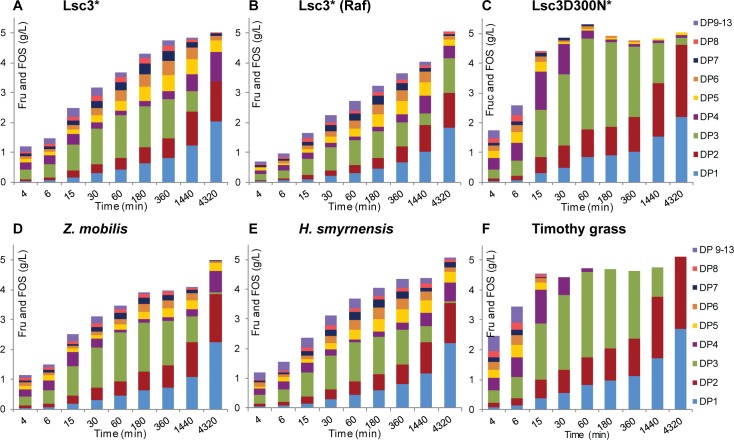
**Time course of degradation of six different levans by the BT1760 into products of varied DP (A-F).** Levans synthesized by Lsc3 or its mutant Asp300Asn (D300N) are designated by an asterisk. Reaction products were analysed using HPLC. Up to four parallel samples were analysed to calculate the average values and standard deviations. Details of the reaction conditions and used methods are presented in Materials and Methods section.

To evaluate capacity and kinetics of FOS production by BT1760, we monitored its 72-h reaction on six different levans added at 5 g/L. Two of the levans (of Lsc3Asp300Asn and timothy grass) had a low molecular weight, two of them (of timothy grass and *Halomonas smyrnensis*) were reported as unbranched, and the Lsc3-produced levan had a very high molecular weight (Table 1). Levan degradation was assayed at 37°C in McIllvaine’s buffer (pH 6.0), HPLC and TLC were used to detect and quantify the reaction products. Results are shown on Figs 7 and 8. The data on FOS yields and DP range of the products at reaction conditions yielding the highest amount of FOS are presented in Table 3.

**Table 3:** Parameters of FOS production by endo-levanase BT1760 from six different levans at optimal reaction duration.

Source of levan	DP range of FOS produced during the optimal^#^ reaction time	Optimal^#^ reaction time for FOS production (min)	Levan conversion to FOS (%) at optimal^#^ reaction time	FOS yield (mg/mg protein) at optimal^#^ reaction time
Lsc3*	2–11	360	78.8 ± 0.9	857.0 ± 9.7
Lsc3* **(Raf)**	2–8	4320	63.0 ± 0.5	684.4 ± 5.8
Lsc3D300N*	2–7	60	91.2 ± 7.4	951.0 ± 80.8
***Zymomonas mobilis***	2–9	180	65.5 ± 0.2	712.3 ± 2.0
***Halomonas smyrnensis***	2–12	360	77.3 ± 1.6	759.4 ± 13.6
**Timothy grass**	2–9	15	69.9 ± 1.3	903.8 ± 48.8

^#^ Optimal reaction time is duration of levan hydrolysis yielding the highest amount of FOS under applied conditions: endo-levanase BT1760 (4.6 μg/mL) was reacted at 37°C with 5 g/L of levans in McIllvaine’s buffer (pH 6.0).

* Levansucrase (Lsc3) originates from *Pseudomonas syringae* pv. tomato. Details of the method and calculations are presented in Materials and Methods section.

The BT1760-assisted FOS production was the highest from Lsc3Asp300Asn and timothy grass levans (Table 3, Figs 7 and 8). TLC start zones on panels C and F of Fig 7 show that levan spot disappeared already by 15 min of the reaction indicating complete degradation of the polymer into FOS. The highest amount of FOS, 4.56 g/L, was recorded at 60 min of endo-levanase reaction on Lsc3Asp300Asn levan. The DP3 FOS formed majority of reaction products (Fig 8C) and the DP7 FOS were the longest (Table 3). In further reaction, the DP3 fraction was hydrolysed to DP2 FOS and fructose (Fig 8C). In good accordance with our data, the DP2 FOS (proven as levanbiose) was the main product of prolonged degradation of levan by *Bacillus licheniformis* LevB1 [34]. FOS release was the most rapid from the timothy grass levan–already by 15 min of the reaction over 4 g/L of total FOS (DP from 2 to 9) were produced (Fig 8F, Table 3). Again, later on the amount of fructose increased at the expense of FOS. Regarding the other four levans used in our assay, the HMW levan produced by Lsc3 from sucrose also yielded a considerably high amount of FOS– 3.94 g/L (DP up to 11) at optimal reaction time, 6 h (Fig 8A, Table 3). The FOS yields were slightly less in the case of Z*ymomonas mobilis* and *Halomonas smyrnensis* levans, and the lowest amount of FOS was produced from raffinose-derived levan (Table 3). The latter was also most resistant to degradation by BT1760 (Figs 7B and 8B) requiring a 72 h reaction time for maximal FOS yield (Table 3).

We could not measure the molecular weight of raffinose-derived levan, but it cannot be of low molecular weight as was thoroughly dialysed using the membrane with MWCO of 12–14 kDa. Kinetic constants of BT1760 for levans synthesized by Lsc3 from sucrose or raffinose were similar (Table 2). We expect that levans produced by Lsc3 from sucrose or raffinose are both of high molecular weight. We also suggest that they are branched which is a typical feature of bacterial levans [31,34,69]. Notably, Tanaka *et al*. [69] have shown that increase of molecular weight of a levan is achieved by increasing the number of branches. Levans of *Erwinia herbicola* and *Zymomonas mobilis* have a moderate molecular weight (Table 1) and similar level of branching [70]. Most interestingly, the *Halomonas smyrnensis* levan which is of moderate molecular weight (Table 1), is characterized as unbranched [63]. According to our knowledge, this is the first example of an unbranched levan of bacterial origin. Our data (Figs 6–8; Tables 2 and 3) show that degradation characteristics of the *Halomonas* levan by the BT1760 endo-levanase are rather similar to those of branched levans with moderate molecular weight.

Fig 7 shows that release of fructose from levans by BT1760 accelerates only at prolonged reaction. This fact once again confirms that the BT1760 is a true endo-levanase and does not conduct exo-cleavage of the fructan. Fig 7 also shows that BT1760 prefers longer FOS to shorter ones regarding their further hydrolysis. In good accordance with this fact, Adamberg *et al*. [10] showed that at growth of *Bacteroides thetaiotaomicron* on levansucrase-produced FOS, long-chain FOS were consumed first by the bacterium. Our data suggest that most probably levanbiose (DP2) is not a substrate for the BT1760 because it accumulates during the reaction (Fig 8). At degradation of LMW levans, the long-chain FOS (of DP 9–13) are detected only at onset of the endo-levanase reaction (Fig 8C and 8F) whilst in the case of other four levans with moderate or high molecular weight, they are present even after 24 h (1440 min) of the reaction.

The highest levan conversion rate to FOS (91.2%) under our experimental conditions was recorded for the Lsc3Asp300Asn levan (Table 3). This value is similar to respective maximum value (97%) reported for the LevB1 endo-levanase of *Bacillus licheniformis* [34]. Even though the FOS production from timothy grass levan was exteremely rapid, the maximum levan conversion rate to FOS was lower (approximately 70%) due to elevated production of fructose. We assume that at degradation of LMW unbranched levan (e.g. timothy grass levan), accession of the endo-levanase to the chain ends is facilitated if compared to HMW branched levans that not only enhances catalytic activity (see Table 1), but also affects the product spectrum.

Earlier, degradation of timothy grass levan by a bacterial endo-levanase (from a rumen bacterium *Butyrivibrio fibrisolvens* 3071) has been studied by Kasperowicz *et al*. [25]. They showed that the *Butyrivibrio fibrisolvens* enzyme degrades timothy grass levan with maximum acivity (V_max_) of 4 U/mg which is comparable to the rate of hydrolysis of LMW bacterial levan by endo-levanases of bacilli [34]. Our data show that the maximum velocity of BT1760 on timothy grass levan is over two hundred times higher indicating much more efficient catalysis. Still, the *Butyrivibrio fibrisolvens* endo-levanase has a good affinity to timothy grass levan, 2.82 g/L [25], that should contribute to efficient degradation of this fructan in the rumen.

### Levans withstand acidic conditions and moist heat sterilization

**Fig 9:**
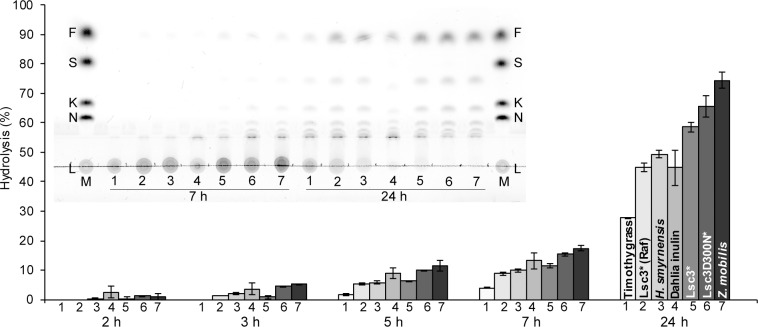
Acid-resistance of levans and dahlia inulin. Fructans (10 g/L) were incubated in 0.01 M hydrochloric acid (pH 2.0) at 37°C for 24 h and sampled over this period for reducing sugar and thin layer chromatography (TLC) analysis. The bars indicate the extent of hydrolysis of the fructan calculated as percentage of released reducing sugars from the total amount of reducing sugars in completely hydrolysed sample. Standard deviation values shown on bars are calculated from at least two independent experiments with at least two parallel samples. TLC shows fructan hydrolysis products at 7 and 24 h of incubation. Samples spotted onto a TLC plate are numbered from 1 to 7 according to respective numbers on the bar diagramme. The chromatogram was developed in chloroform: acetic acid: water (60:70:10; v/v/v). Sugar markers (M) on TLC: 7 g/L levan (L), 8 mM nystose (N), 8 mM 1-kestose (K), 30 mM sucrose (S) and 30 mM fructose (F). Details of the methods are presented in Materials and Methods section.

In general, β-linked fructans are considered resistant to gastric acid and human digestive enzymes [71–72]. Thereby they can reach the colon undegraded and serve as a selective food for colon microbiota. Yet, some publications refer to moderate hydrolysis of β-linked fructans by gastric acid [73]. Level of pH in the stomach of healthy adults is 1.5–3.5, whereas in children under the age of two years and in elderly people the stomach acidity decreases [74–76]. We assayed the resistance of levans to hydrolysis by 0.01 M hydrochloric acid (pH 2.0) at 37°C mimicking conditions in the stomach. Dahlia inulin was studied as a β-2,1 linked reference fructan. Production of reducing sugars from fructans was monitored during 24 h of incubation and spectrum of produced products was analysed using TLC. Fig 9 shows that all studied fructans are acid-stable. By 2 h of incubation in 0.01 M HCl, less than 2% of reducing sugar was released from the polymers. Up to 7 hours of incubation, the level of hydrolysis stayed relatively low. After 7 h of incubation, a low amount of fructose and FOS can be seen on a TLC plate. At 24 h of incubation, degradation of polymeric fructan (see disappearance of a non-migrating spot at the start zone of the TLC plate in Fig 9) was the highest in the case of dahlia inulin and levans of Lsc3Asp300Asn and *Zymomonas mobilis*. Timothy grass levan showed lowest extent of degradation. It is not clear why timothy grass levan has the highest stability at acidic conditions. Interestingly, this assay also shows that levan synthesized from raffinose by Lsc3 withstands acid hydrolysis better than levan synthesized from sucrose.

Levan-derived FOS have to be stable at stomach acidity conditions if applied as prebiotics. Our data show that FOS produced from levan with endo-levanase of *B*. *thetaiotaomicron* are sufficiently acid-stable–only after 4 h of incubation of the FOS at pH 2.0, a fructose spot emerged on a TLC plate. From 4 to 7 h of treatment the amount of fructose and short-chain FOS increased at the expense of longer ones (S4 Fig).

To study prebiotic properties of fructans, growth of bacterial pure cultures and gut consortia on these substrates should be studied using sterile growth media. Threfore we studied degradation-resistance of 10 g/L fructan solutions in mQ water to moist heat-sterilization in the autoclave at two temperatures: 112°C and 121°C. After autoclaving, the amount of reducing sugar was measured in the samples and TLC assay was also carried out. No degradation of fructans due to autoclaving was observed.

### What happens to levan in the gut and to the gut if levan is present?

Several studies suggest that presence of levan in the colon can have a protective and health-promoting effect on colonocytes. A recent *in vitro* assay by Bondarenko *et al*. [67] showed that Lsc3-produced levan had no harmful effect on metabolic activity and integrity of the Caco-2 cells. Moreover, if mineral nanoparticles were coated with respective levan, their toxic effect on Caco-2 cells was reduced (in the case of Se) or totally eliminated (in the case of Co_3_O_4_). Two different assays unequivocally demonstrated that levan enhanced the metabolic activity of the Caco-2 cells, but did not induce cellular proliferation [67]. Concordant to these results, levan from a halophilic bacterium *Halomonas* sp. (it was used as one of endo-levanase substrates in current study) did not affect proliferation of osteoblasts and murine macrophages *in vitro* and protected a marine crustacean *Artemia salina* from chemical-induced toxicity [36]. Transepithelial electrical resistance measurements of levan-exposed Caco-2 cells suggested that levan entered the cellular membranes, stabilized them providing unspecific protection [67].

Aside of protecting colonocytes, levan and levan-type FOS are potentially prebiotic–numerous strains of probiotic bacteria, bifidobacteria and lactobacilli, can grow on either levan or levan-type FOS [33–34,77]. Levan utilization by a gut commensal *Bacteroides thetaiotaomicron* was first reported by Sonnenburg *et al*. [7]. Adamberg *et al*. [10] showed that *B*. *thetaiotaomicron* ferments Lsc3-produced levan to SCFA (mostly acetic, D-lactic, propionic and succinic acids were detected). If growth of fecal consortia on Lsc3-produced levan was addressed by the same group [11], acetic, lactic, butyric, propionic, succinic acids and carbon dioxide were detected as the main excreted metabolites. Association between the growth of levan-degrading (e.g. *Bacteroides*) and butyric acid-producing (e.g. *Faecalibacterium*) bacteria was detected in the fecal consortia suggesting feeding of butyrate-producing bacteria on levan-derived metabolites [11]. Butyrate production in the gut is important–it is the main energy source for colonocytes, inhibits proliferation of colon cancer cells and induces their apoptosis [78–79]. Cross-feeding hypothesis by Adamberg *et al*. [11] agrees with data by Rakoff-Nahoum *et al*. [12] who showed that when grown on levan, *B*. *thetaiotaomicron* releases levan breakdown products (FOS and fructose) into the medium to be consumed by other gut symbionts.

Food containing levan is common for some regions of the Earth. So, a traditional Japanese food natto, prepared by fermentation of soybeans, contains levan as *Bacillus subtilis* used in the fermentation produces levan from soybean sugars [31,80]. Though natto is considered a health-promoting and age-prolonging product, it is not a trivial food constituent in most regions of the Earth, including Europe. Yet, there are certain fructan-containing food sources in Europe, for example cereals. As cited in Verspreet *et al*. [28], most cereal fructans belong to graminans and their concentration in the grain dry weight comprises up to 6.5% (in the case of rye) and 2.9% (in the case of wheat). We assume that endo-levanases of gut bacteria facilitate degradation of cereal graminans. A report by Jenkins and coworkers [81] indicates that barley grain graminans are *in vitro* fermented by human fecal slurry microbiota resulting in SCFA production comparable to that observed with commercial inulin-type FOS preparation. Jensen *et al*. [55] showed that the *Bacillus subtilis* endo-levanase LevB partly degrades plant (ryegrass) graminans.

Our data presented and discussed here suggest that food containing levan, phlein or graminan, or supplemented with these polymers, should serve as prebiotic for *B*. *thetaiotaomicron* that has both exo- and endo-acting levan-degrading enzymes. As several gut *Bacteroides* species such as *B*. *caccae*, *B*. *vulgatus* and others are capable of growth on levan breakdown products [12], multiplication of these species should be also promoted by these substrates. In addition, levan can stimulate growth of other gut bacteria such as *Bacteroides xylanisolvens* and *Butyrivibrio fibrisolvens*, which were predicted by us to degrade levan. Importantly, one strain of *B*. *xylanisolvens* isolated from human gut has already been confirmed as safe and without virulence potential [82]. While *Bacteroides* species does not produce butyrate as fermentation product [10], *Butyrivibrio* species do [78]. Besides levan, *Butyrivibrio fibrisolvens* also degrades starch and xylan, cellulose and inulin [25,83]. The above-mentioned bacteria could be considered potential probiotics which can be selectively stimulated by levan. Our study showed that a plant levan (from timothy grass) is a very good substrate for the endo-levanase of *B*. *thetaiotaomicron*. To enable prebiotic efficiency studies, plant levans should be available in sufficiently high amounts. Herein it should be noted that a highly promising transgenic production system of timothy levan in sugar beet expressing timothy 6-SFT genes was recently presented by Matsuhira *et al*. [84].

### Outlook

We do hope that this study arises new interest towards levans as their physiological effects are much less studied than those of another polymeric fructan–inulin. We report here that all six different levans studied by us, including the levan of plant origin, are degraded into fructooligosaccharides by the endo-levanase BT1760 –a cell surface protein of an abundant gut resident *Bacteroides thetaiotaomicron*. Therefore, levans have a high potential to selectively stimulate multiplication and metabolic activity of this bacterium in the gut, and also supply co-inhabiting bacteria with levan degradation products. Hopefully, further studies of gut microbiota will disclose also other beneficial bacterial species that can use levan. As most of the levans are branched and can have extremely high molecular weights, they are ‘difficult’ substrates for endo-acting hydrolases. Therefore, detailed study of endo-levanases should be an intriguing challenge for enzymologists and structural biologists to determine the structure elements responsible for the substrate binding and specificity.

## Supporting Information

S1 Fig The Lsc3 protein is an extremely stable catalyst. (PDF)

S2 Fig Overexpression of the endo-levanase BT1760 in *Escherichia coli*. (PDF)

S3 Fig The effect of pH on levan-degrading activity of the endo-levanase BT1760. (PDF)

S4 Fig Acid-stability of fructooligosaccharides (FOS) produced by hydrolysis of levan with the endo-levanase BT1760. (PDF)

S1 Table Accession numbers of protein and gene sequences analysed in current work. (PDF)
