# Supplementary material for: A Highly Active Endo-Levanase BT1760 of a Dominant Mammalian Gut Commensal Bacteroides thetaiotaomicron Cleaves Not Only Various Bacterial Levans, but Also Levan of Timothy Grass
Source: PLoS One. 2017 Jan 19;12(1):e0169989. doi: 10.1371/journal.pone.0169989 (PMC5245892; doi:10.1371/journal.pone.0169989)
Supplement: S1 Fig — (PDF) [file pone.0169989.s001.pdf]

A Highly Active Endo-Levanase BT1760 of a Dominant Mammalian Gut Commensal *Bacteroides thetaiotaomicron* Cleaves Not Only Various Bacterial Levans, but Also Levan of Timothy Grass

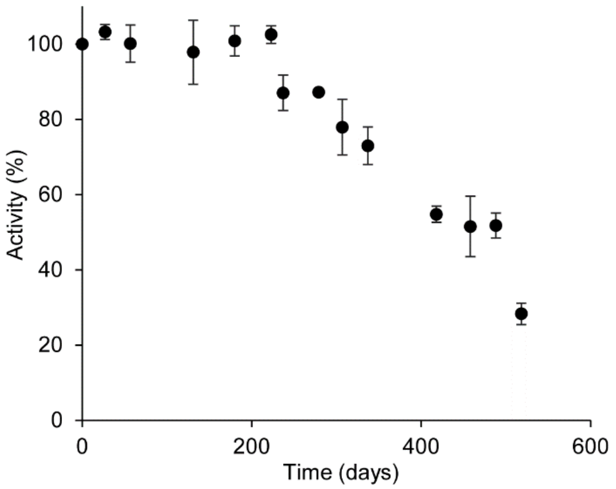

S1 Fig. The Lsc3 protein is an extremely stable catalyst.
Sucrose-splitting activity of Lsc3 preparation kept at 37°C for 500 days was monitored as shown in Materials and Methods section of the main text. Percentage from initial activity of the enzyme is plotted against incubation time and the mean ± standard deviation values of three parallel measurements are shown.
