# Supplementary material for: A Highly Active Endo-Levanase BT1760 of a Dominant Mammalian Gut Commensal Bacteroides thetaiotaomicron Cleaves Not Only Various Bacterial Levans, but Also Levan of Timothy Grass
Source: PLoS One. 2017 Jan 19;12(1):e0169989. doi: 10.1371/journal.pone.0169989 (PMC5245892; doi:10.1371/journal.pone.0169989)
Supplement: S2 Fig — (PDF) [file pone.0169989.s002.pdf]

A Highly Active Endo-Levanase BT1760 of a Dominant Mammalian Gut Commensal *Bacteroides thetaiotaomicron* Cleaves Not Only Various Bacterial Levans, but Also Levan of Timothy Grass

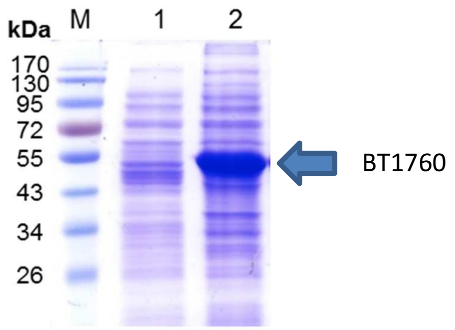

S2 Fig. Overexpression of the endo-levanase BT1760 in *Escherichia coli*.
Lysates of *E. coli* BL21(DE3) (10 µg of total protein) carrying either the empty vector (pURI3Cter; lane 1) or the pURI3-BT1760Cter vector (lane 2) were analyzed by SDS-PAGE. The endo-levanase BT760 with the C-terminal His_6 tag has the molecular weight of 58 kDa. M – PageRuler^{TM} prestained protein ladder (Thermo Scientific, USA) was loaded as a size-reference.
