# Supplementary material for: A Highly Active Endo-Levanase BT1760 of a Dominant Mammalian Gut Commensal Bacteroides thetaiotaomicron Cleaves Not Only Various Bacterial Levans, but Also Levan of Timothy Grass
Source: PLoS One. 2017 Jan 19;12(1):e0169989. doi: 10.1371/journal.pone.0169989 (PMC5245892; doi:10.1371/journal.pone.0169989)
Supplement: S3 Fig — (PDF) [file pone.0169989.s003.pdf]

A Highly Active Endo-Levanase BT1760 of a Dominant Mammalian Gut Commensal *Bacteroides thetaiotaomicron* Cleaves Not Only Various Bacterial Levans, but Also Levan of Timothy Grass

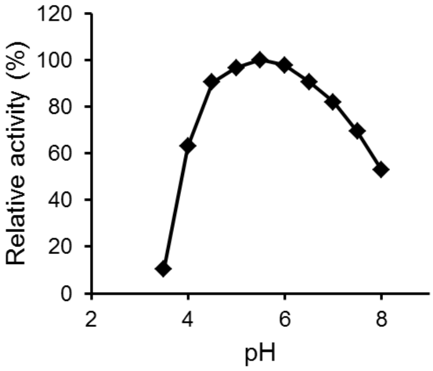

S3 Fig. The effect of pH on levan-degrading activity of the endo-levanase BT1760.
Degradation of Lsc3-produced levan (5 g/L) was measured in McIllvaine's buffers of varied pH. Reaction was conducted in microplate wells at 37°C and initial rate of turbidity decrease at 400 nm was recorded using a Tecan infinite M200 PRO^{TM} reader (Tecan Group Ltd., Switzerland). The final volume of reaction mixture in the wells was 100 µl and 3 µg/mL of endo-levanase protein was added to start the reaction. Initial velocities of the reaction are presented as decrease of turbidity per minute per mg of protein. 100% of activity corresponded to 47.4 ± 5.0 U/mg, measured at 37°C and pH 5.5. The average values were calculated from at least three parallel samples. Standard deviation values did not exceed 10%.
