# Supplementary material for: A Highly Active Endo-Levanase BT1760 of a Dominant Mammalian Gut Commensal Bacteroides thetaiotaomicron Cleaves Not Only Various Bacterial Levans, but Also Levan of Timothy Grass
Source: PLoS One. 2017 Jan 19;12(1):e0169989. doi: 10.1371/journal.pone.0169989 (PMC5245892; doi:10.1371/journal.pone.0169989)
Supplement: S4 Fig — (PDF) [file pone.0169989.s004.pdf]

A Highly Active Endo-Levanase BT1760 of a Dominant Mammalian Gut Commensal *Bacteroides thetaiotaomicron* Cleaves Not Only Various Bacterial Levans, but Also Levan of Timothy Grass

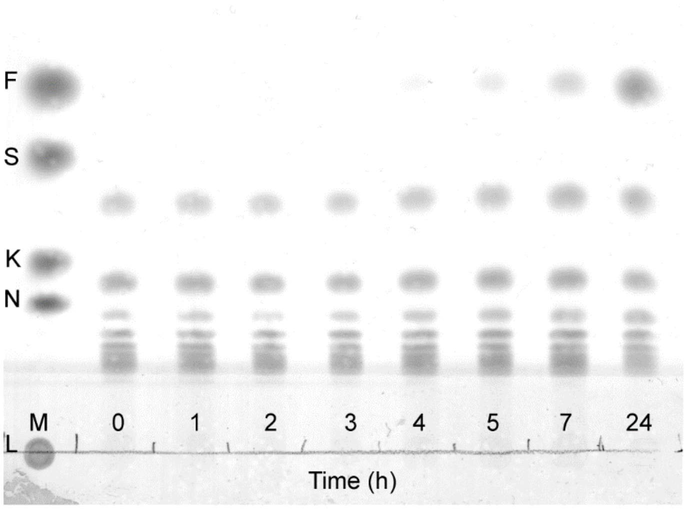

S4 Fig. Acid-stability of fructooligosaccharides (FOS) produced by hydrolysis of levan with the endo-levanase BT1760.

The FOS (13 g/L) were incubated in 0.01 M hydrochloric acid (pH 2.0) for up to 24 h, samples were withdrawn at times shown on the figure, neutralized and spotted onto a thin layer chromatography (TLC) plate. The chromatogram was developed with chloroform: acetic acid: water (60:70:10; v/v/v). Sugar markers (M): levan (L), nystose (N), 1-kestose (K), sucrose (S) and fructose (F). Details of FOS production from levan and TLC conditions are given in Materials and Methods section of the main text.
