# Supplementary material for: A Highly Active Endo-Levanase BT1760 of a Dominant Mammalian Gut Commensal Bacteroides thetaiotaomicron Cleaves Not Only Various Bacterial Levans, but Also Levan of Timothy Grass
Source: PLoS One. 2017 Jan 19;12(1):e0169989. doi: 10.1371/journal.pone.0169989 (PMC5245892; doi:10.1371/journal.pone.0169989)
Supplement: S1 Table — (PDF) [file pone.0169989.s005.pdf]

A Highly Active Endo-Levanase BT1760 of a Dominant Mammalian Gut Commensal *Bacteroides thetaiotaomicron* Cleaves Not Only Various Bacterial Levans, but Also Levan of Timothy Grass

S1 Table. Accession numbers of protein and gene sequences analysed in current work

Protein (locus) designation	Protein (locus) characterization	Microorganism	Accession No	
			UniProtKB	GenBank
Lsc3 (Lsc-3)	Levansucrase#	*Pseudomonas syringae* pv. tomato DC3000	Q88BN6	AAO59056.1
BT1760	Endo-levanase*	*Bacteroides thetaiotaomicron* VPI-5482	Q8A6W6	AAO76867.1
BT1754	Hybrid two-component sensing and regulatory system (HTCS)*	*Bacteroides thetaiotaomicron* VPI-5482	Q8A6X1	AAO76861.1
BT1757	Fructokinase*	*Bacteroides thetaiotaomicron* VPI-5482	Q8A6W9	AAO76864.1
BT1758	Inner membrane monosaccharide importer*	*Bacteroides thetaiotaomicron* VPI-5482	Q8A6W8	AAO76865.1
BT1759	Exo-acting fructanase*	*Bacteroides thetaiotaomicron* VPI-5482	Q8A6W7	AAO76866.1
BT1761	Protein specifically binding levan*	*Bacteroides thetaiotaomicron* VPI-5482	Q8A6W5	AAO76868.1
BT1762	Levan-specific SusD homologue*	*Bacteroides thetaiotaomicron* VPI-5482	Q8A6W4	AAO76869.1
BT1763	SusC homologue*	*Bacteroides thetaiotaomicron* VPI-5482	Q8A6W3	AAO76870.1
BT1765	Exo-acting fructanase*	*Bacteroides thetaiotaomicron* VPI-5482	Q8A6W1	AAO76872.1
BT3082	Exo-acting fructanase*	*Bacteroides thetaiotaomicron* VPI-5482	Q8A373	AAO78188.1
Genomic region (76953-98008 nt, reverse); supercontig 1.5, whole genome shotgun sequence	Putative fructan PUL	*Bacteroides xylanisolvens* CL03T12C04		NZ_JH724298.1
HMPREF1074_04233	Homologous to BT1760	*Bacteroides xylanisolvens* CL03T12C04	I9UP90	EIY84278.1
HMPREF1074_04239	Homologous to BT1754	*Bacteroides xylanisolvens* CL03T12C04	I9JCE0	EIY84284.1
HMPREF1074_04236	Homologous to BT1757	*Bacteroides xylanisolvens* CL03T12C04	I9UNP0	EIY84281.1
HMPREF1074_04235	Homologous to BT1758	*Bacteroides xylanisolvens* CL03T12C04	I9A953	EIY84280.1
HMPREF1074_04234	Homologous to BT1759	*Bacteroides xylanisolvens* CL03T12C04	I9JCD5	EIY84279.1
HMPREF1074_04232	Homologous to BT1761	*Bacteroides xylanisolvens* CL03T12C04	I9AAC9	EIY84277.1
HMPREF1074_04231	Homologous to BT1762	*Bacteroides xylanisolvens* CL03T12C04	I9UNN7	EIY84276.1
HMPREF1074_04230	Homologous to BT1763	*Bacteroides xylanisolvens* CL03T12C04	I9A947	EIY84275.1
HMPREF1074_04228	Homologous to BT1765	*Bacteroides xylanisolvens* CL03T12C04	I9UP85	EIY84273.1
LevB1	Endo-levanase	*Bacillus licheniformis* strain IBT-ALM	W8GV60	AHK25455.1
LevB	Endo-levanase	*Bacillus subtilis* 168	O07003	CAB15451.1
	Endo-levanase	*Butyrivibrio fibrisolvens* 16/4	D4IW69	CBK75012.1
INU2	Endo-inulinase	*Aspergillus ficuum*	O94220	AJ006951.1

#Characterized in refs [22,40 and 45] of the main text.

*Function addressed in Sonnenburg et al. (2010), ref [7] of the main text.
